# Antimicrobial Usage and Detection of Multidrug-Resistant *Staphylococcus aureus*: Methicillin- and Tetracycline-Resistant Strains in Raw Milk of Lactating Dairy Cattle

**DOI:** 10.3390/antibiotics12040673

**Published:** 2023-03-29

**Authors:** Lubna, Tahir Hussain, Ashwag Shami, Naseem Rafiq, Shehryar Khan, Muhammad Kabir, Naimat Ullah Khan, Irfan Khattak, Mustafa Kamal, Tahir Usman

**Affiliations:** 1 College of Veterinary Sciences and Animal Husbandry, Abdul Wali Khan University Mardan, Mardan 23200, Pakistan; 2 Department of Microbiology, Abdul Wali Khan University Mardan, Mardan 23200, Pakistan; 3 Department of Biology, College of Sciences, Princess Nourah bint Abdulrahman University, P.O. Box 84428, Riyadh 11671, Saudi Arabia; 4 Department of Zoology, Abdul Wali Khan University Mardan, Mardan 23200, Pakistan; 5 Department of Biotechnology, Abdul Wali Khan University Mardan, Mardan 23200, Pakistan; 6 Department of Biological Sciences, Thal University Bhakkar, University of Sargodha, (Ex-Sub Campus Bhakkar), Bhakkar 30000, Punjab, Pakistan

**Keywords:** antimicrobial resistance, *Staphylococcus aureus*, mastitis, resistance genes, tetracycline, methicillin

## Abstract

*Staphylococcus aureus* is a prominent cause of food-borne diseases worldwide. Enterotoxigenic strains of this bacteria are frequently found in raw milk, and some of these strains are resistant to antimicrobials, posing a risk to consumers. The main objectives of this study were to determine the antimicrobial resistance pattern of *S. aureus* in raw milk and to detect the presence of *mecA* and *tetK* genes in it. A total of 150 milk samples were obtained aseptically from lactating cattle, including Holstein Friesian, Achai, and Jersey breeds, maintained at different dairy farms. The milk samples were checked for the presence of *S. aureus*, and it was detected in 55 (37%) of them. The presence of *S. aureus* was verified by culturing on selective media, gram staining, and performing coagulase and catalase tests. Further confirmation was performed through PCR with a species-specific thermonuclease (*nuc*) gene. Antimicrobial susceptibility testing of the confirmed *S. aureus* was then determined by using the Kirby–Bauer disc diffusion technique. Out of the 55 confirmed *S. aureus* isolates, 11 were determined to be multidrug-resistant (MDR). The highest resistance was found to penicillin (100%) and oxacillin (100%), followed by tetracycline (72.72%), amikacin (27.27%), sulfamethoxazole/trimethoprim (18.18%), tobramycin (18.18%), and gentamycin (9.09%). Amoxicillin and ciprofloxacin were found to be susceptible (100%). Out of 11 MDR *S. aureus* isolates, the methicillin resistance gene (*mecA*) was detected in 9 isolates, while the tetracycline resistance gene (*tetK*) was found in 7 isolates. The presence of these methicillin- and tetracycline-resistant strains in raw milk poses a major risk to public health, as they can cause food poisoning outbreaks that can spread rapidly through populations. Our study concludes that out of nine empirically used antibiotics, amoxicillin, ciprofloxacin, and gentamicin were highly effective against *S. aureus* compared to penicillin, oxacillin, and tetracycline.

## 1. Introduction

Bovine mastitis is an inflammation of udder tissue caused mainly by microbial infection or by physical trauma, resulting in enormous economic losses to the dairy industry [1]. Mastitis affects approximately 40% of cows and costs the world economy EUR 125 billion every year [2,3]. Mastitis manifests in two forms: clinical or subclinical. Clinical mastitis (CM) is characterized by sudden onset, change in milk composition, a reduction in milk production, and the appearance of basic symptoms of inflammation in the infected udder that can be easily identified. Sometimes, clinical mastitis is characterized by fever and decreased appetite [4], whereas in subclinical mastitis (SCM), no obvious symptoms are detected in the udder or milk. However, the output of milk decreases and somatic cells increase [5]. Subclinical mastitis occurs more frequently than CM. Both types of mastitis result in changes in milk composition of varying degrees, depending on the severity of the disease [6]. The milk in CM is rejected by the farmer, owing to obvious abnormalities, whereas SCM causes no visible alterations and is, thus, included in bulk milk [7].

Mastitis can affect human health by promoting the spread of bacteria that are resistant to antibiotics, which is worrisome [8]. Despite considerable research and attention devoted to controlling mastitis, it remains one of the most expensive diseases affecting dairy cattle [9]. Milk from infected cows is unfit for human use due to bacterial contamination [10]. Mastitis is a global issue because it endangers not only the health of cattle but also human lives by contaminating the milk with pathogens and lowering the production of milk, ultimately resulting in significant economic losses [11].

*S. aureus* is one of the main bacteria that cause mastitis in dairy cattle [12]. It is a gram-positive, facultative anaerobic bacterium that is oxidase-negative, catalase-positive, and coagulase-positive coccus. *S. aureus* is spherical in shape, 0.5–1.5 µm in diameter, non-spore-forming, and non-motile. It is a major infectious agent for both the community and the health sector [13,14]. In 1881, Alexander Ogaston, a Scottish surgeon, first identified *S. aureus* in a surgical abscess [15]. Besides infecting people and animals, *S. aureus* also colonizes pets and wild species, where it may behave as an opportunistic pathogen [16]. *S. aureus* is a serious pathogen that can cause intramammary infection in dairy animals. According to reports, it causes 40% of cases of mastitis in various countries [17,18,19]. *S. aureus* has the ability to develop resistance and generates a variety of virulence elements, including endotoxins and other hazardous proteins. It causes recurring and chronic infections and is highly resistant to β-lactam antibiotics. Its rate of resistance has dramatically increased in recent years [20].

*S. aureus* has been recognized as a major source of zoonotic infections as well as a transmission factor of methicillin-resistant *S. aureus* (MRSA) from animals to humans through bodily interaction, handling, or ingestion of *S. aureus*-contaminated animal products [21]. *S. aureus* contamination of raw milk and infection of dairy cattle, particularly when it has a phenotype of MDR and is capable of forming biofilm and toxins, such as TSST-1, PVL, and enterotoxin, continues to be a major concern for the health of people [22]. Food-borne poisoning incidents linked to *S. aureus*-contaminated dairy products, involving one of the worst outbreaks of food-borne diseases ever recorded in Japan involving 13,420 infected people, show the bacterium’s significance for public health [23]. Consumption of unprocessed milk is a primary source of *S. aureus* infections, which can cause common and severe illnesses, including septicemia, pneumonia, and dermatitis in humans [24]. Various antimicrobial agents are used on farm animals on a daily basis as a therapy against *S. aureus* mastitis or to enhance production, which results in the emergence of MDR and MRSA among farm animals.

Antimicrobial resistance (AMR) has been highlighted as a serious danger to world health. According to the World Health Organization (WHO), mutations in bacteria can lead to AMR, making drugs ineffective and causing illnesses to remain in the body, increasing the risk of dissemination to others [25]. There are various explanations underlying the development of AMR, ranging from microbial origins to human components, such as the misuse and over-prescription of antimicrobial drugs, the agricultural and manufacturing application of antimicrobials in the animal sector, and human behavioral factors [26]. MDR *S. aureus* infections have been linked to a number of infectious diseases, a high rate of morbidity, and financial loss to the dairy industry. MRSA, which is linked with animals and communities, was found recently, and it represents a major public health issue [27].

Antimicrobials are frequently used to treat microbial diseases in farm animals, such as cattle [28]. However, due to lateral gene transfer, the prevalence of antibiotic-resistant genes in the *Staphylococcus* species is a major problem, as these resistant bacterial strains can be carried from animals to humans [29]. The *mecA* gene (methicillin), *tetM/tetK* (tetracycline), *blaz* gene (methicillin), and *ermA/ermC* gene (erythromycin), as well as *msrA/msrB*, are common antibiotic resistance genes in the dairy sector that have been reported worldwide [30]. Mastitis is often treated with an intramuscular or intravenous injection of antibiotics, such as ampicillin, streptomycin, cloxacillin, tetracycline, and penicillin. However, due to the fast development of antibiotic-resistant bacteria, the therapy is expected to become troublesome in the near future [31]. As a result, other alternatives to antibiotic therapy must be sought.

Using antimicrobials for a long time might cause the formation of bacterial strains that are antimicrobial-resistant, which is a major worry not only for animal welfare but, more crucially, for human health as well. The purpose of this study was to find out the prevalence and antimicrobial resistance of the genes *mecA* and *tetK* among *S. aureus* isolates, which were isolated from raw milk collected from dairy farms in Khyber Pakhtunkhwa, Pakistan.

## 2. Results

### 2.1. Somatic Cell Count

The samples were divided into three groups based on SCC. The number of cattle in SCC Group 1 (≤200,000 cells/mL of milk, healthy cows) was 72, of which 38 were Achai, 24 were Jersey, and 10 were Holstein Friesian. SCC Group 2 (>200,000–500,000 cells/mL of milk, infected cows) included 27 cattle, of which 4 were Achai, 9 were Jersey, and 14 were Holstein Friesian, while SCC Group 3 (>500,000 cells/mL of milk, severely infected cows) had 51 cattle, of which 19 were Achai, 7 were Jersey, and 25 were Holstein Friesian.

### 2.2. Prevalence of S. Aureus

A total of 150 milk samples were screened for the detection of *S. aureus*. A total of 55 (37%) milk samples were found positive. The presence of *S. aureus* was verified by culturing on selective media, gram staining, and coagulase and catalase tests. The catalase and coagulase positivity and the purple round shape colonies observed under a microscope confirmed the presence of *S. aureus*. *S. aureus* was further confirmed molecularly through PCR with a species-specific thermonuclease (*nuc*) gene.

The breed-wise prevalence of *S. aureus* was recorded highest in Holstein Friesian (48.98%) followed by Achai (37.70%), while the lowest prevalence was recorded in Jersey (20%). The prevalence difference between the different cattle breeds was statistically significant (*p* < 0.05). *S. aureus* prevalence was highest in the Harichand dairy farm at 65.30%, followed by the Munda dairy farm, 41.66%; Field 1 Dir, 28.57%; and the Agricultural University Peshawar dairy farm, 28%. The lowest prevalence was recorded in Hanifa Research Center Dir (8.69%), whereas 0% prevalence was recorded in Field 2 Swat sampling (Table 1). S. aureus prevalence had a significant association with the farm of cattle (*p* < 0.05).

### 2.3. Antibiotic Susceptibility Test

An antibiotic susceptibility test (AST) was carried out on all *S. aureus* isolates (*n* = 55). All confirmed *S. aureus* isolates were grouped according to the Clinical and Laboratory Standards Institute (CLSI) guideline 2018 as resistant, intermediate, and susceptible for each antimicrobial drug tested. The highest resistance was found to penicillin (100%) and oxacillin (100%), followed by tetracycline (72.72%), amikacin (27.27%), sulfamethoxazole/trimethoprim (18.18%), tobramycin (18.18%), and gentamycin (9.09%). On the other hand, amoxicillin and ciprofloxacin were found to be 100% susceptible (Table 2). Out of the 55 verified *S. aureus* isolates, 11 were determined to be MDR.

### 2.4. Detection of Resistance Genes

All *S. aureus* MDR isolates were screened for resistance genes, i.e., *mecA* and *tetK*, through PCR. The methicillin resistance gene (*mecA*) was detected in 9 isolates out of 11, whereas the tetracycline resistance gene (*tetK*) was found in 7 isolates out of 11. The amplified gene sizes were 533 bp and 360 bp for *mecA* and *tetK*, respectively.

## 3. Discussion

In the present study, *S. aureus* was found in 37% of milk samples collected from different breeds of cattle, including Holstein Friesian, Jersey, and Achai. The high prevalence is due to the poor hygienic environment in the farms during milk processing. The results of the present study are in line with previous studies that reported nearly similar prevalences of *S. aureus* in milk samples in China, Italy, and Brazil [28,32,33]. A recent study conducted in Khyber Pakhtunkhwa, Pakistan, reported a 34% prevalence of staphylococcal mastitis in lactating bovine [12]. Another study conducted by Bano et al. [34] in three provinces (Khyber Pakhtunkhwa, Punjab, and Sindh) of Pakistan found a 45% prevalence of *S. aureus* in raw milk. Aqib et al. [35] carried out similar research in Faisalabad, Pakistan, and concluded that 34% of the raw milk samples were infected with *S. aureus*. Other studies from Pakistan and Iran reported 34.2%, 80.79%, 37.14%, and 12.4% prevalences of *S. aureus* [28,36,37,38].

In the present study, the difference in *S. aureus* prevalence between different cattle breeds (Holstein Friesian, Achai, and Jersey) was statistically significant (*p* < 0.05). The highest prevalence was reported in the exotic Holstein Friesian breed. The variation in *S. aureus* occurrence between breeds may be due to the inherited characteristics, immunity of different breeds, and cows’ habituation to the impact of climate [39]. In the present study, *S. aureus* prevalence had a significant association with the farm of cattle (*p* < 0.05). The difference in *S. aureus* prevalence in cattle farms is associated with the contamination in the housing facilities of the cattle and teat exposure to the surrounding environment [39].

In this study, the in vitro disc sensitivity tests show that the resistance of *S. aureus* was highest to penicillin (100%) and oxacillin (100%), followed by tetracycline (72.72%), amikacin (27.27%), sulfamethoxazole/trimethoprim, and tobramycin (18.18%). Gentamycin was proven to have less resistance (9.09%). Isolates, on the other hand, were more susceptible to amoxicillin (100%) and ciprofloxacin (100%) and least to penicillin and oxacillin (0%). The antibiogram revealed that eleven of the total isolates (20%) were MDR. The probable development of resistance through the extended and indiscriminate use of some antimicrobials is suggested by Stefani and Guglio [40]. Correspondingly, a report by Beyene [41] in Ethiopia revealed that all isolates of *S. aureus* were resistant to penicillin in cow milk. The subclinical mastitis isolates of *S. aureus* in German dairy cows also proved to be 74.28% resistant to Penicillin [42]. In Brazilian mastitis buffalo, *S. aureus*-resistant isolates of oxacillin have been discovered in 50% of isolates [43].

Recent investigations have revealed high frequencies of *S. aureus* resistance to penicillin and tetracycline in unpasteurized milk. In Iran, according to the results of Jamali et al. [28], strains isolated from bovine raw milk were found resistant to penicillin and tetracycline at 44.4% and 56.2%, respectively. Conversely, Gao et al. [44] conducted similar research in China and reported that 96.2% of *S. aureus* raw milk isolates are penicillin-resistant and 98.1% are tetracycline-resistant.

The treatment of *S. aureus* infection in cattle is widespread. In various countries, farmers routinely use penicillin and tetracycline in dairy cattle to eliminate *S. aureus* infections from the herd [45]. Penicillin and tetracycline resistance increases with persistent and pervasive usage due to breed, nutrition, and climate [28,45]. Tetracycline resistance, especially from the *tetK* gene, was mostly dependent on the efflux mechanism of staphylococci. This conclusion is consistent with prior veterinarian observations. The *tetK* gene from tetracycline was the most often identified in staphylococci resistance [46]. Surprisingly, *S. aureus* isolates were discovered in China to be significantly tetracycline-resistant (98.1%) in a single herd [44]. While in India, tetracycline resistance was found in 57% of dairy cows (Pati and Mukherjee, 2016). Saidi et al. [47] also found that *S. aureus* isolates from bovine mastitis in Algeria had a rather high (61.9%) resistance to tetracycline.

MRSA is a major issue in both animals and humans. In the current study, 9 (81.81%) out of 11 MDR *S. aureus* isolates were found to be MRSA, with the *mecA* gene present. Aqib et al. [35] from Faisalabad, Pakistan, reported a lower (34%) prevalence of MRSA in cattle and buffaloes. In the neighboring country, India, Ganai et al. [48] reported that 44.1% of the *S. aureus* isolates were MRSA. Other studies conducted in Turkey, Egypt, China, and Algeria reported 75.4%, 35.7%, 9.61%, and 4.1% prevalences of MRSA [49,50,51,52]. The main causes of increased MRSA prevalence are the irregular use of beta-lactam antibiotics and poor hygienic conditions during milking [35].

## 4. Materials and Methods

### 4.1. Study Design and Sample Collection

The study was carried out on selected pure breeds of cattle, including Achai (A), Holstein Friesian (HF), and Jersey (J), maintained at Khyber Pakhtunkhwa dairy cattle farms. A total of 150 samples, including HF (*n* = 49), Jersey (*n* = 40), and Achai (*n* = 61), were collected. Aseptic methods were used in the collection of milk to prevent contamination by bacteria present on the skin, udder, teat, and farm environment. The udder was cleaned and disinfected with ethanol (70%), and the first few streaks were discarded from each teat canal. Milk samples were collected in 15 mL pre-labeled sterile milk bottles with 0.01 mg of potassium dichromate as a preservative and transferred in an ice box (at 4 °C) to the laboratory for further biological processes.

### 4.2. Somatic Cell Count

Microscopic slides were prepared from the milk samples and examined for somatic cell count (SCC) under a microscope by using the protocol of Usman et al. [53]. Somatic cell count (SCC) was categorized into three major groups based on somatic cells/mL of milk: healthy animals (SCC, <200,000 cells/mL of milk), elevated SCC ranged between 200,000 to 500,000 cells/mL of milk, whereas the high SCC group had SCC higher than 500,000 cells/mL of milk.

### 4.3. Bacterial Isolation

*S. aureus* was isolated and identified by directly streaking milk onto mannitol salt agar (MSA), then incubated at 37 °C for 24–48 h. The bacteriological media were prepared according to the protocol of Quinn et al. [54]. The plates were analyzed for the growth of *S. aureus* colonies. For pure cultivation, staphylococcal colonies were sub-cultured and incubated at 37 °C for 24–48 h on freshly prepared nutrient agar. The development of presumed colonies was determined by utilizing traditional bacteriological techniques based on colony, pigment production, and hemolysis features.

### 4.4. Biochemical Tests

The isolated *S. aureus* was further confirmed by using different biochemical tests, i.e., gram staining (+coccus) and catalase (+) and coagulase tests, as described by Quinn et al. [25]. For the catalase test, *S. aureus* pure colonies that were between 18 and 24 h old were selected. A single colony was picked and placed on a glass slide using a sterile inoculating loop. Using a dropper, 3 percent hydrogen peroxide solution was poured on the glass slide. Immediate bubbles were observed, which were considered positive results, while no bubble formation was considered a negative test result. To distinguish *S. aureus* from the rest of the staphylococcal species, a coagulase test was practiced. On a clean, glass slide, an *S. aureus* colony was mixed in a drop of water, making a smear. A sterile wire loop was dipped into the plasma, and the attached plasma traces were added to the suspension of *Staphylococcus* created on the slide. When a bacterial suspension and plasma are combined, the coagulase enzyme that *S. aureus* generates causes the cells to clump.

### 4.5. S. aureus Stock Preparation

Brain–heart infusion (BHI) was prepared according to the manufacturer’s protocol. Fresh *S. aureus* colonies from NAP were added to BHI media and incubated for 24 h at 37 °C. Turbidity showed growth of *S. aureus* after incubation time. For stock preparation, 50 μL of glycerol was taken in microcentrifuge tubes (Eppendorf), and 1 mL BHI (containing *S. aureus*) was added to it and stored at −80 °C until further use.

### 4.6. DNA Extraction and Molecular Identification of S. aureus

DNA was extracted from the samples by using the protocol that was previously established by Walsh et al. [55]. A 5% Sigma-Aldrich Chelex^®^ 100 stock solution was prepared by adding 5 g of Chelex powder and 95 mL of water followed by proper vertexing. An amount of 200 microliters of the 5% Chelex was pipetted out into a 1.5 mL Eppendorf tube, and several bacterial colonies were picked by sterile wire loop and dipped into Chelex, which was followed by mixing multiple times using slow pipetting to avoid bubbling. The reagent was then incubated at a temperature of 65 °C for 8 min. The reagent was vortexed several times to ensure Chelex encountered the bacterial colonies to extract DNA. The reagent was again heated at 60 °C for 7 min and, after cooling, centrifuged at 12,000 rpm for 2 min. The supernatant was collected into separate tubes, as it contained DNA, while the pellet was discarded, as it had the remaining Chelex reagent. The collected DNA was stored at −4 °C for further use.

The *S. aureus* isolates were molecularly identified through PCR, with a species-specific thermonuclease (*nuc*) gene, as previously described by Louie et al. [56]. The primers used in the amplification of the *nuc* gene are given in Table 3.

### 4.7. Antimicrobial Susceptibility Testing

The *S. aureus* isolates isolated from milk were examined in vitro for their susceptibility to various antimicrobial agents frequently used in veterinary practices. Using the Kirby–Bauer disc diffusion test technique on Muller–Hinton agar (MHA), the antibiotic susceptibility of *S. aureus* isolates was evaluated [57]. Antimicrobial agents (Oxoid, Basingstoke, UK) including penicillin (10 μg), tetracycline (30 μg), oxacillin (10 μg), amoxicillin (30 μg), ciprofloxacin (10 μg), amikacin (30 μg), gentamycin (10 μg), sulfamethoxazole/trimethoprim, and tobramycin (10 μg) were used.

Colonies obtained from pure culture were placed in a glass tube containing 5 mL of broth culture, a colony suspension was prepared, and the colony was incubated at 37 °C for 8 h. The turbidity of the isolates’ direct colony suspensions was adjusted, in comparison to the turbidity corresponding to a 0.5 McFarland standard, for the antimicrobial drugs determined for isolated strains using the disc diffusion method. To prepare the Muller–Hunter agar (MHA) plates, one colony of mannitol salt agar was picked up with a cotton swab and diluted in 3 mL of normal saline in a glass tube. Bacterial lawns were then formed through the cotton bud (swab). The MHA agar plate was covered with these bacteria lawns such that not a single point remained empty. After lawn preparation, antibiotic discs were applied aseptically. Following that, the plates were incubated at 37 °C for 24 h.

According to Clinical and Laboratory Standards Institute (CLSI) criteria, the inhibition zone was measured. The Krumperman [58] approach was used to calculate the Multiple Antibiotic Resistance (MAR) Index. The bacterial isolates were acknowledged as having multidrug resistance (MDR) when they were found resistant to three or more categories of antimicrobial drugs, as defined by Magiorakos et al. [59].

**Table 3:** Primers for the amplification of *nuc*, *mecA* and *tetK* genes of *S. aureus*.

Genes	Nucleotide Sequence	Base Pair	Reference
*Nuc*	Forward: 5′ GCGATTGATGGTGATACGGTT 3′ Reverse: 3′ CGAAATCAAGCAGTTCCGAACCGA 5′	270	[60]
*MecA*	Forward: 5′ AAAATCGATGGTAAAGGTTGGC 3′ Reverse: 3′ AGTTCTGCAGTACCGGATTTGC 5′	533	[61]
*TetK*	Forward: 5′ GTAGCGACAATAGGTAATAGT 3′ Reverse: 3′ GTAGTGACAATAAACCTCCTA 5′	360	[62]

### 4.8. Molecular Detection of Antimicrobial Resistance Gene

A well-established methodology was utilized to identify the antibiotic resistance genes, i.e., *mecA* and *tetK*. Polymerase chain reaction was employed to amplify and identify the necessary resistance genes by utilizing primers (Table 3), and the reaction was performed in a thermal cycler. A 20 µL reaction was produced by adding 10 μL of master mix, 2 µL of DNA sample, 6 µL of PCR-grade water, and 1 µL (10 μM Conc) of each of the forward and reverse primers. The thermal cycling procedure for PCR was initial denaturation at a temperature of 95 °C for 10 min and a 2nd denaturation temperature of 95 °C for 30 s, and the annealing temperature was held at 55 °C for 30 s and polymerization at 72 °C for 30 s, with a final extension phase at 72 °C for 10 min for a total of 35 cycles. The PCR product was visualized on 2 percent agarose gel using gel electrophoresis.

### 4.9. Gel Electrophoresis

The amplified PCR product was subjected to gel electrophoresis. To make 2 percent agarose gel, 0.40 g of agarose gel was added to 20 mL of TAE buffer in a small beaker. The mixture was swirled to blend. The agarose/buffer mixture was melted by heating it in the microwave for 30 s at a time and swirling it until the agarose was completely dissolved. Ethidium bromide (EtBr) was added to a concentration of 0.5 μg/mL solution after it had been appropriately brought to a boil. A 15-tooth comb was placed in a casting tray to create wells. For appropriate solidification, the gel was put onto the tray and allowed to sit at room temperature for 20 min. The 1st well of gel was loaded with 4 μL of 1 kb ladder marker. Afterward, 5 μL of the PCR product was added to the other wells in order to conduct the assessment. For gel electrophoresis, gels containing TAE buffer and electrodes connected to the negative and positive terminals were exposed to 120 volts for 30 min at 500 mA current. The gel was removed from the gel tray and exposed to UV light by using a gel documentation system that shows orange fluorescent DNA bands.

### 4.10. Statistical Analysis

The data from well-established government dairy farms were collected and transferred to excel sheets. A statistical study was undertaken to determine the relationship between *S. aureus* prevalence and different cattle breeds and farms. The chi-squared test was used, and *p* < 0.05 was considered significant.

## 5. Conclusions

This study revealed that out of the 55 *S. aureus*-positive isolates, 11 isolates showed multidrug resistance. Out of 11 MDR *S. aureus* isolates, the methicillin resistance gene (*mecA*) was detected in 9 isolates, while the tetracycline resistance gene (*tetK*) was found in 7 isolates. The presence of these methicillin- and tetracycline-resistant strains in raw milk poses a major risk to public health, as these strains can induce food poisoning outbreaks that spread across populations. This study concludes that among the nine empirically used antibiotics, amoxicillin, ciprofloxacin, and gentamicin were highly effective against mastitis compared to penicillin, oxacillin, and tetracycline. This study infers that dairy farms should use only those antibiotics to which bacteria are sensitive. It is recommended that farm workers should be aware of adequate antibiotic usage in dairy farms because resistant strains among dairy cows may pose a danger to human health due to the possibility of ingesting contaminated food, mainly raw milk.

## Figures and Tables

**Table 1:** Prevalence of *S. aureus* in different farms and breeds of cattle.

Variable	Prevalence % (Infected/Total)	Chi-Square (χ^2^)	*p*-Value
**Farm**			
Harichand dairy farm	65.30 (32/49)	32.97	0.0001 *
Agriculture University Peshawar dairy farm	28% (7/25)		
Munda dairy farm	41.66% (10/26)		
Hanifa research center Dir	8.69% (2/23)		
Field 1 Dir	28.57% (4/14)		
Field 2 Swat	0% (0/13)		
**Breed**			
Holstein Friesian	48.98% (24/49)	8.08	0.018 *
Jersey	20% (8/40)		
Achai	37.70% (23/61)		

* = *p* < 0.05.

**Table 2:** Antimicrobial susceptibility of *S. aureus* isolates.

Antimicrobial Agents	Conc (μg)	Zone Diameter (mm)		
		Sensitive %	Intermediate %	Resistant %
Oxacillin	10	≥13 (0%)	11–12 (0%)	≤10 (100%)
Gentamycin	10	≥15 (72.72%)	13–14 (18.19%)	≤12 (9.09%)
Tetracycline	30	≥19 (27.28%)	15–18 (0%)	≤14 (72.72)
Ciprofloxacin	10	≥21 (100%)	16–20 (0%)	≤15 (0%)
Penicillin	10	≥29 (0%)	- (0%)	≤28 (100%)
Amoxicillin	30	≥20 (100%)	- (0%)	≤19 (0%)
Sulfamethoxazole/ trimethoprim	25	≥16 (81.82%)	11–15 (0%)	≤10 (18.18%)
Tobramycin	10	≥15 (72.73%)	13–14 (9.09%)	≤12 (18.18%)
Amikacin	30	≥17 (63.64%)	15–16 (9.09%)	≤14 (27.27%)

## Data Availability

All relevant data are within the manuscript.
